# Mechanical Behavior and Structural Characterization of a Cu-Al-Ni-Based Shape-Memory Alloy Subjected to Isothermal Uniaxial Megaplastic Compression

**DOI:** 10.3390/ma15103713

**Published:** 2022-05-22

**Authors:** Vladimir Pushin, Nataliya Kuranova, Alexey E. Svirid, Yurii Ustyugov

**Affiliations:** Laboratory of Non-Ferrous Alloys, M.N. Mikheev Institute of Metal Physics of Ural Branch of Russian Academy of Sciences, 620108 Ekaterinburg, Russia; kuranova@imp.uran.ru (N.K.); svirid2491@rambler.ru (A.E.S.); ustyugov@imp.uran.ru (Y.U.)

**Keywords:** copper alloys, quenching, megaplastic compression, thermoelastic martensitic transformation, shape-memory effect, physical and mechanical properties, fine-grained structure

## Abstract

For the first time, uniaxial megaplastic compression was successfully applied to a polycrystalline shape-memory Cu-Al-Ni-based alloy. The samples before and after uniaxial megaplastic compression were examined by methods of X-ray diffraction, optical, electron transmission, and scanning microscopy. The temperature dependences of electrical resistance and the mechanical properties of the alloys under uniaxial tension were also measured. The mechanical behavior under uniaxial megaplastic compression in isothermal conditions in the range of 300–1073 K was studied using the Instron 8862 electric testing machine. The microstructure, phase composition, and martensitic transformations in the eutectoid alloy (Cu-14wt.%Al–4 wt.%Ni) were studied. The radical refinement of the grain structure of the initial hardened *D*0_3_ austenite was found under controlled isothermal compression, due to dynamic recrystallization in the temperature range 673–1073 K and velocities of 0.5–5 mm/min. Compression at 873–1073 K was accompanied by simultaneous partial pro-eutectoid decomposition with the precipitation of the γ_2_ phase. Compression at temperatures of 673 and 773 K—that is, below the eutectoid decomposition temperature (840 K)—was accompanied by the precipitation of disperse γ_2_ and α phases, and ultradisperse *B*2’ particles. Cooling of the deformed alloy to room temperature after performing each regime of compression led to thermoelastic martensitic transformation, together with the precipitation of the β′ and γ′ phases. The formation of a fine-grained structure produced an unusual combination of strength and plasticity of the initially brittle alloy both under controlled uniaxial compression, and during subsequent tensile tests at room temperature.

## 1. Introduction

Diffusionless martensitic transformations (MTs) are the most common among those that occur in metallic materials. Being deformation-induced phase transitions of the first (I) kind, with significant effects of specific volume changes (Δ*V*/*V*), they provide high strength, plasticity, and other operational characteristics of such widely used structural materials as steels and iron-based alloys [1]. These mechanical properties in steels are mainly due to MTs caused by the high number and density of dislocations inside highly disperse crystals of α-martensite, morphologically and orientationally distributed in accordance with the orientation relationships (O.S.) close to the O.S. of Kurdjumov and Sachs [2]. At the same time, the so-called thermoelastic martensitic transformations (TMTs), which are seen in various predominantly non-ferrous alloys, are not accompanied by irreversible plastic deformation, due to their much lower values of Δ*V*/*V* and differences in the crystal lattices of austenite and martensite, and can be completely reversible [3,4,5,6]. TMTs are responsible for a number of other unusual and extremely important physical phenomena [3,4,5,6,7,8,9]. It is obvious that the single or multiple cyclically reversible shape-memory (SM) effects, high values of superelasticity (GS), damping, and many other effects due to TMTs distinguish intelligent or smart alloys into a special separate class of new, practically important, structural and multifunctional application-employed materials [3,4,5,6,7,8,9,10,11,12].

It has recently been discovered that upon direct or reverse TMTs, such unique caloric effects as magnetocaloric, barocaloric, and elastocaloric effects are also observed depending on a variety of external influences (e.g., thermal, force-acting, electrical, magnetic), when the former are in demand only in effective and innovative heating and refrigeration technologies [13,14,15,16,17,18,19,20,21,22,23,24,25,26]. Therefore, first of all, alloys based on titanium nickelide, and characterized by a unique set of mechanical properties and SM effects, have found wide application in engineering and medicine [3,4,5,6,7,8,9,10,11,12].

Among SM alloys, copper β-alloys of the Cu-Al-Ni, Cu-Zn-Al, Cu-Zn-Sn, and other systems attract attention owing to their much lower cost, better thermal and electrical conductivity, and processability, even compared to the best fine-grained titanium nickelide alloys [3,4,5]. These alloys, when in the single-crystal state, show excellent characteristics. However, in their usual coarse-grained (CG) state, these polycrystalline alloys exhibit very low characteristics of ductility, fatigue durability, and fracture toughness. [4,27]. This prevents the SM effects inherent to their single crystals from being achieved. In conditions of high elastic anisotropy, a certain TMT becomes useless for producing SM effects, due to the generated catastrophic brittleness of the alloys. The physical nature of this phenomenon consists of the progressive accumulation of coherent elastic stresses in the course of MT, due to the increase in the absolute magnitude of the volumetric effect |Δ*V*/*V*|. In this case, the only significant localization of elastic stresses in polycrystalline alloys has to occur within the grain boundaries of a general type. The smaller their length, and the higher the level of elastic stresses (as well as, consequently, brittleness), the larger the grain sizes. As is known, copper β-alloys in the pre-martensitic state experience a strong softening of the elastic modulus *C′* and an increase in elastic anisotropy A, which are associated with their grain boundary brittleness [8,28]. Moreover, an additional important reason that prevents the employment of these aging alloys or the eutectoid SM alloys is the fact of their coarse-grained structure and intercrystalline brittleness, as a consequence of grain boundary decomposition. Therefore, it seems natural to develop technologies that ensure the refinement of grains to increase the plasticity and prevent the brittleness of these alloys.

Previously, it was found that the fine- and ultrafine-grained (FG and UFG) structures lead to an improvement in the strength, plasticity, and fatigue characteristics of SM alloys based on titanium nickelide [29,30,31,32,33,34,35,36,37,38,39,40,41,42,43]. The UFG structure of Ti-Ni alloys is provided by various thermal deformation technologies using megaplastic deformation (MPD) methods, including multi-pass equal-channel angular pressing (ECAP), rolling, and drawing to strips, rods, or wire. In our works [29,44,45,46,47,48,49,50,51], a significant reduction in the embrittlement of copper SM alloys was found due to a radical decrease in grain size under MPD, and an increase in the lengths of their boundaries. Various other methods using alloying additives, rapid quenching, heat treatment, powder metallurgy, and a number of other methods have not been successful, and did not provide a noticeable refinement of the grain structure of these alloys or improve their plasticity [27].

It was also shown that warm ECAP, in contrast to other MPD methods that are predominantly deformable by structural mechanism, is most preferable for the formation of a UFG structure, due to the predominance of the dynamic recrystallization mechanism [29,30,31,32,33,34,35,36,37,38,39]. Another similar method of grain-size refinement of the grain structure of titanium alloys is warm abc-pressing [52]. We have shown that uniaxial hot-pressing can also be effective for refinement of the grain structure in CG copper alloys [46,49,50]. The aim of this work is to study the mechanical behavior, structural features, and phase composition of metastable SM Cu-Al-Ni alloys upon using uniaxial compression technology.

## 2. Materials and Methods

A 60 mm diameter ingot of the three-component master alloy Cu-14Al-4Ni was made by electric arc melting from high-purity Cu, Al, and Ni (99.99%). The chemical composition of the alloy, as determined by spectral analysis with a Bruker Q4 Tasman Spectrometer, was 13.95 wt.% Al, 4.02 wt.% Ni, and Cu the remainder. The alloy was homogenized in the furnace in an atmosphere of purified helium at 1173 ± 25 K, for 8 h. Then, after heating to 1223 K for 30 min, the ingot was forged into bars with a cross-section of 20 mm × 20 mm. After reheating at 1223 K for 10 min, the alloy was quenched in water at room temperature (RT). Uniaxial megaplastic compression (MPC) of the alloy at various temperatures and deformation rates was carried out using the Instron 8862 electromechanical measuring system, equipped with an electric furnace for deformation under isothermal conditions at temperatures up to 1073 K, on cylindrical blanks with a diameter (*d*_0_) of 7.5 mm and a height (*h*_0_) of 9.2 mm. To prevent the solid-solution decomposition, the samples after MPC were quenched in water at RT. Mechanical tensile tests were performed on Instron test machines—namely, 5982 (on standard samples with *d*_0_ = 3 mm) and 3545 (on flat samples with a working part of 1 × 0.2 × 3 mm)—at RT. Structural-phase studies were performed using optical microscopy (OM) and scanning electron microscopy (SEM) with a Quanta-200 Pegasus microscope (at 30 kV), and transmission electron microscopy (TEM) with a Tecnai G^2^ 30 microscope (at 300 kV), as well as X-ray diffraction (XRD) analysis with a Bruker D8 Advance diffractometer in monochromatized Cu Kα radiation. When measuring the temperature dependences of electrical resistance ρ(*T*), the critical temperatures of the start (*M_s_*, *A_s_*) and finish (*M_f_*, *A_f_*) of the forward and reverse TMTs were determined by the tangent method.

## 3. Results

**Figure 1:**
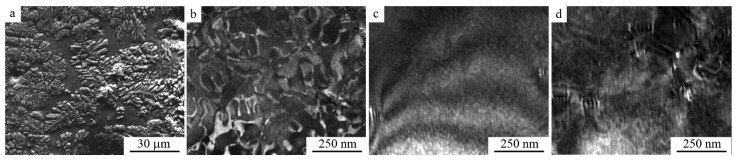
(**a**) Secondary electron SEM image and (**b**,**d**) bright- and (**c**) dark-field TEM images (amplitude contrast) of the Cu-14Al-4Ni alloy in the (**a**) as-cast and (**b**–**d**) quenched austenite states.

It has been established that the studied cast-and-forged β-alloy under subsequent cooling in air undergoes decomposition according to the scheme β→β_1_ + γ_2_ (at temperatures above the temperature of eutectoid decomposition *T*_ED_, equal to 840 K), and eutectoid decomposition β_1_→α + γ_2_ (at temperatures below *T*_ED_) (Figure 1a), according to [4,7]. The average grain size in the CG alloy was close to 1 mm. According to the OM data, taking into account the size measurements of 200 grains, grain size varied within 0.5–1.5 mm. Quenching after hot forging prevents eutectoid decomposition. Figure 1b,c show TEM images typical of the intragrain microstructure of β_1_-austenite. Since β-austenite, when cooled above *T*_ED_ and M_s_, can experience two consecutive “disorder-order” phase transitions (β→β_2_(*B*2)→β_1_(*D*0_3_)) [4], a special substructure of so-called anti-phase domains (AFDs) is formed, where AFDs are visualized by the appearance of their boundaries (AFBs) seen on dark-field TEM images in superstructural reflections (Figure 1b). In this case, the long-range atomic order of the initial ordered austenitic phase is inherited by the structure of martensite (*M*), which determines its (*M*) important role in TMT in the realization of the effects of the orientational crystal–structural reversibility and phase thermoelasticity [4,5]. In the TEM study, a pre-martensitic tweed diffraction contrast was also observed on bright- and dark-field (in structural reflections) images of austenite (Figure 1c,d). The tweed contrast was naturally extinguished under certain diffraction conditions set by the inclination of the samples in the goniometer. The tweed contrast was oriented along various crystallographic directions which, as the analysis showed, were mainly intersections of the lattice planes {110} with the foil surface [51]. This contrast had a thin substructure formed by the equiaxed and lamellar elements of the alternating homogeneous contrast (AHC), the dimensions of which depended on the diffraction conditions. Against the background of the AHC, the contrast from the dislocations and AFBs was visible. The contrast and dimensions of the tweed elements on the AFBs were greater, indicating the heterogeneous localization of these elements along these coherent superstructural sub-boundaries (Figure 1d). Such an increase in the tweed contrast was also noted in the images of the dislocations, whether inclined or horizontally disposed.

**Figure 2:**
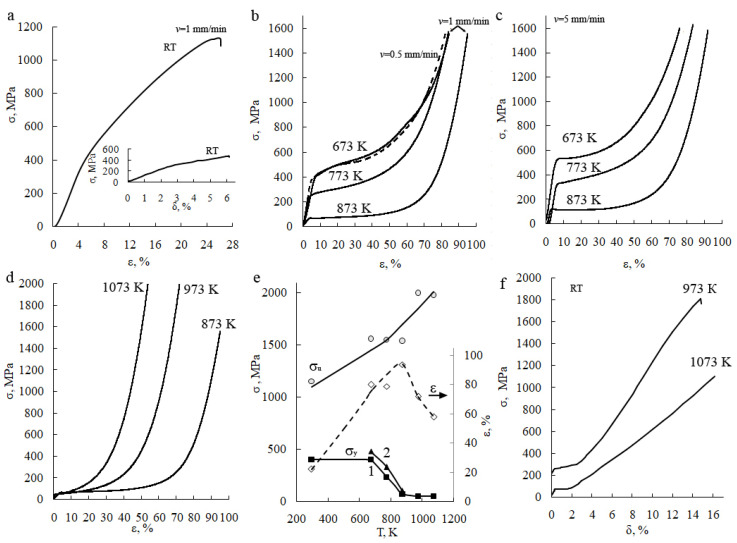
Stress–strain curves for the Cu–14Al–4Ni alloy under compression tests at a rate of (**a**,**b**,**d**) 1 (continuous line), (**b**) 0.5 (dashed line), and (**c**) 5 mm/min, at various temperatures (RT, 673, 773, 873, 973, and 1073 K); (**e**) yield stress σ_y_, ultimate stress σ_u_, and total compression deformation ε vs. temperature *T* at rate of (1) 1 and (2) 5 mm/min; (insert in (**a**)); and (**f**) “σ-δ” curves under subsequent tensile tests at RT after (insert in (**a**)) quenching at 1223 K or (**f**) MPC at temperatures of 973 and 1073 K.

From the results of the mechanical compression tests at RT, it follows that the Cu-14Al-4Ni alloy in its initial state experienced a sufficiently large plastic deformation to fracture—close to ε = 22%—with a high yield stress σ_0.2_ and strength limit σ_u_, whose values were close to 400 and 1150 MPa, respectively (Figure 2a). The σ–ε curve had a classic appearance; four main stages of deformation could be distinguished on it, differing in mechanisms and coefficients of deformation hardening: (1) the stage of elastic deformation; (2) and (3) the stages of deformation hardening, differing in hardening coefficients; and (4) a short stage of localization of deformation, terminating with fracture. In the insert to Figure 2a, the stress–strain curve obtained for this alloy based on its uniaxial tension at RT is also shown for comparison. Comparison of the obtained results shows that at close values of the coefficient of deformation strengthening θ = dσ/dε (~4 and 5 GPa under compression and tensile tests, respectively), the relative elongation δ of the alloy samples to fracture under tension (δ = 4%) is more than five times lower than the value of ε ≈ 22%.

Mechanical tests at elevated temperatures of 673, 773, and 873 K showed that the samples of Cu-14Al-4Ni alloy are capable of experiencing large plastic deformation under uniaxial compression, without failure, up to high values of the strength limit σ_u_, which reached 1600–2000 MPa (Figure 2b–d). The recorded engineering curves “stress σ—deformation ε” had a classic appearance in terms of shape, with four distinct stages of deformation, differing in their mechanisms and coefficients of deformation hardening. It is possible to distinguish the stage of elastic deformation; the stage of light, steady, uniform deformation, which differs depending on the temperature and rate in the values of the yield strength σ_y_ and the strengthening coefficients (θ_1_ = dσ_1_/dε); the transitional stage of rapidly increasing deformation strengthening; and, finally, the stage of strong strengthening (θ_2_ = dσ_1_/dε).

A comparison of the data given in Figure 2b–d and obtained at different compression rates *v* (0.5; 1; 5 mm/min) shows that the values of the deformation hardening coefficient θ_2_ and the accumulated relative compression ε (or its true logarithmic value *e* = ln *h*_0_/*h*_τ_) are quite close, and MPC with a higher rate generally does not lead to higher strength characteristics of σ_u_ (Table 1). At the same time, a stronger influence of the deformation rate and temperature on the stress σ_y_ was found at the beginning of the plastic flow (Figure 2e). Thus, an increase in *v* from 1 mm/min to 5 mm/min led to an increase in the value of σ_y_ from 380 to 530 MPa at a deformation temperature of 673 K, from 250 to 310 MPa at 773 K, and from 70 to 120 MPa at 873 K. This testifies to the predominance of structural deformation hardening processes, with an increase in the rate and a decrease in the deformation temperature compared with compensating softening processes or, on the other hand, progression under compression with an increase in temperature, a decrease in the deformation rate and, accordingly, an increase in the duration τ. Together with an increase in the value of σ_y_, the plasticity of the alloy ε decreased slightly with an increase in the rate and a decrease in the deformation temperature (Table 1). Attention should also be drawn to the growth of σ_u_ and decrease in ε at compression temperatures of 973 and 1073 K, at which the alloy mainly experiences solid-solution hardening without noticeable pro-eutectoid decomposition (Figure 2e).

**Figure 3:**
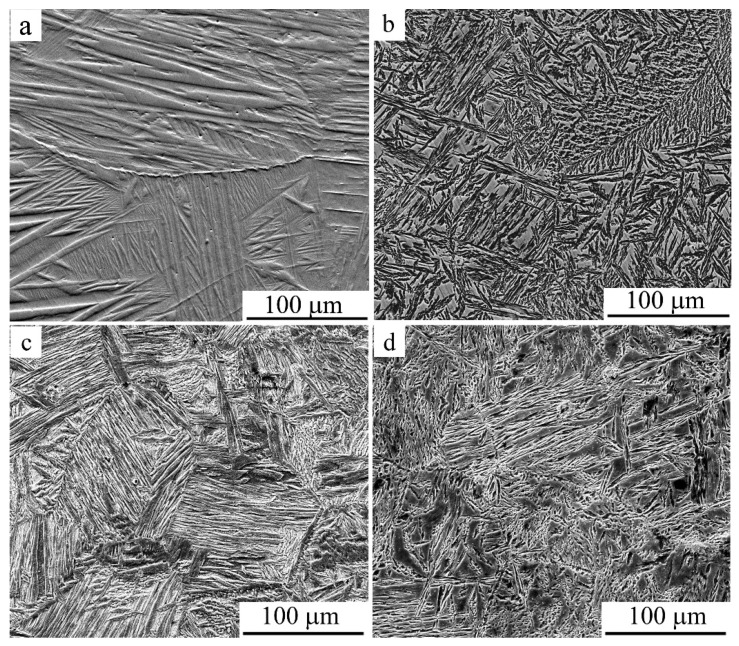
Secondary electron SEM images of the deformation-induced martensite in the Cu-14Al-4Ni alloy after (**a**) tensile tests or (**b**–**d**) compression tests at (**b**) RT and (**c**,**d**) 773 K ((**c**)—*v* = 5 mm/min, (**a**,**b**,**d**)—*v* = 1 mm/min).

**Figure 4:**
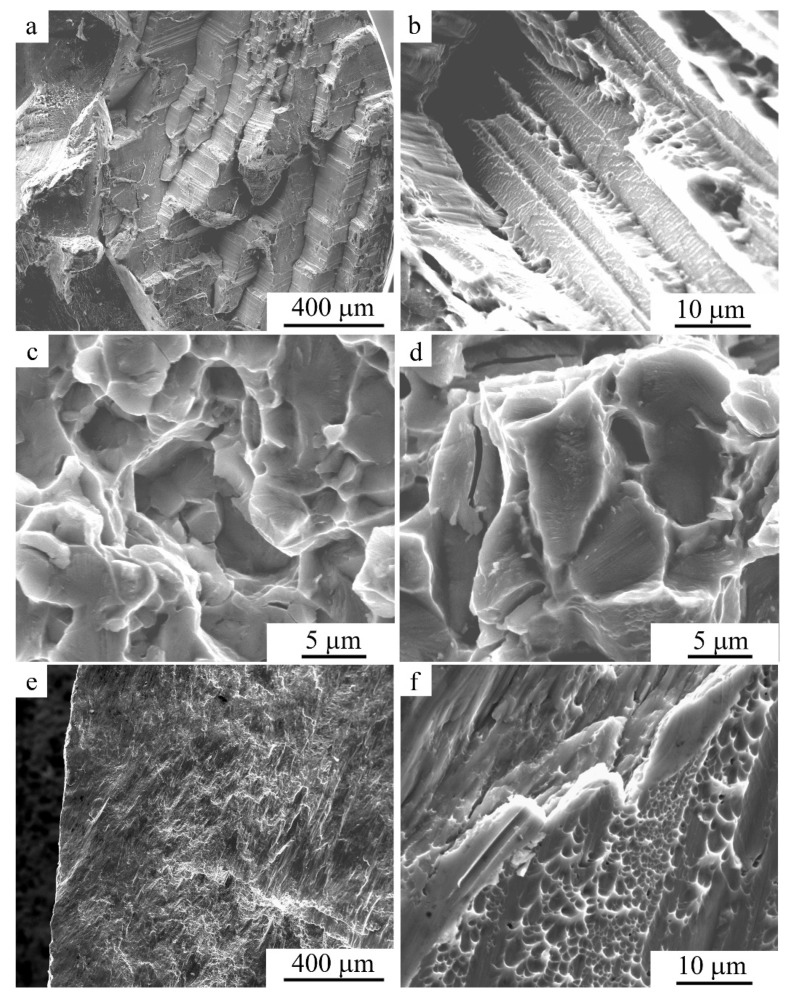
Secondary electron SEM images of the fracture surfaces in the Cu-14Al-4Ni alloy after (**a**–**d**) tensile tests or (**e**,**f**) compression tests.

Figure 3 shows SEM images of a microstructure subjected to special sample preparation by deeper chemical etching of the alloy after tensile (a) and compression (b–d) tests. It can be seen that both tension and compression ensure the formation of a martensitic structure in the alloy at RT. It should be noted that after compression, compared with tension, the morphology changed significantly, and the martensite crystals in the dominant packet-wise morphology were significantly refined in size. Large arch-like joints were virtually not detected, and the sizes of both individual martensite crystals and their packets significantly decreased. Moreover, austenitic grain sizes were also decreased after warm compression (for example, at 773 K; Figure 3c,d). The images typical of the fracture surface, which were obtained for the samples after tension or compression, are shown in Figure 4a–f. It follows from the SEM images that the brittle and tough–brittle intergranular and intragranular examples of the fracture of the martensitic alloy after tensile testing at RT occurred mainly along the boundaries of packets of twin-oriented martensite crystals (Figure 4a,b). When compressing at RT, intrasurface areas with a narrow, dimpled fracture were formed more often, indicating increased intragrain deformability of the alloy, with predominance of a tough–ductile fracture mechanism (Figure 4e,f).

**Figure 5:**
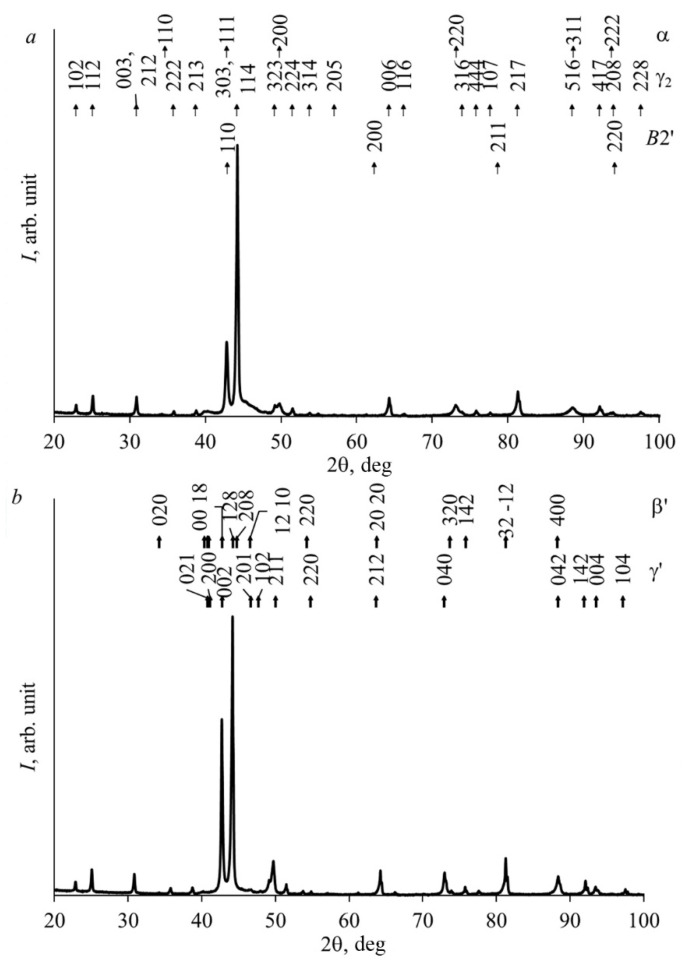
X-ray diffraction patterns for the Cu–14Al–4Ni alloy taken after MPC with *v* = 1 mm/min at (**a**) 673 and (**b**) 873 K.

It is important to note that in the studied alloys below *T*_ED_~840 K, according to the phase equilibrium diagram, eutectoid decomposition of an atomically ordered β_1_(*D*0_3_) solid solution was found to occur [4]. In this case, aluminum-enriched cubic intermetallic Ni-Al–Cu-based phases *B*2′ (lattice parameter *a* is close to 0.289 nm) and γ_2_-Cu_9_Al_4_ of *D*8_3_ -type (*a* is close to 0.870 nm), as well as aluminum-depleted α-*A*1 (FCC; *a* is close to 0.361 nm), were formed. It was obvious that in the Cu-14Al-4Ni alloy, eutectoid decomposition occurred during mechanical compression at elevated temperatures (673–873 K). XRD, SEM, and TEM studies of the samples subjected to MPC were carried out to determine the real changes in the microstructure and phase composition. According to the XRD data, two martensitic phases (β′-type 18*R*: lattice parameters *a* = 0.445 nm, *b* == 0.523 nm, *c* = 3.805 nm, β = 91.0°; and γ′-type 2*H*: lattice parameters *a* = 0.439 nm, *b* = 0.519 nm, *c* = 0.433 nm) were indeed present in the MPC alloy, the precipitates of the γ_2_ phase after compression at 873–1073 K, and also, possibly, the α phases after MPC at 673 and 773 K. Figure 5 shows examples of XRD patterns after MPC at 673 and 873 K. The axial-deformation-induced recrystallization texture of austenite of the type <110>*_D_*_03_ was clearly manifested, inherited by the martensitic phases when cooled to RT, which led to a significant increase in the intensity of the diffraction peaks in the angle range of 2θ ~ 42–45°.

**Figure 6:**
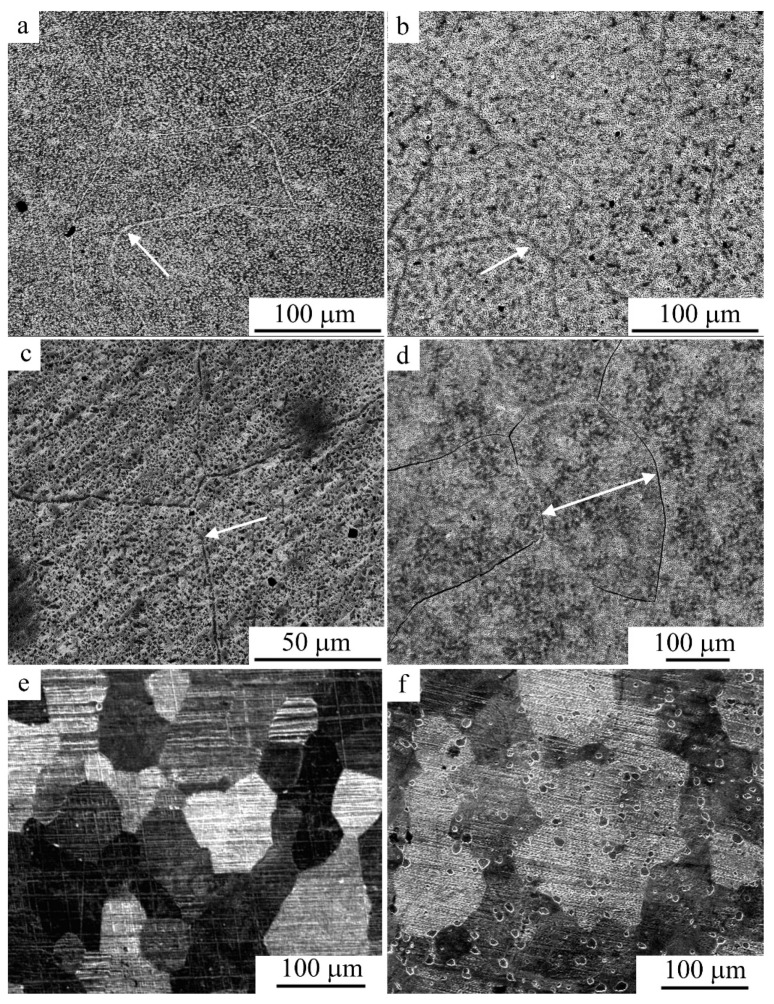
(**a**–**d**) Secondary electron SEM images and (**e**,**f**) OM images of the FG structure in the Cu-14Al-4Ni alloy after the compression tests at (**a**–**c**) 673 K ((**a**)—*v* = 5, (**b**)—*v* = 1, (**c**)—*v* = 0.5 mm/min), (**d**) 873 K (*v* = 5 mm/min), (**e**) 973 K (*v* = 1 mm/min), and (**f**) 1073 K (*v* = 1 mm/min).

**Figure 7:**
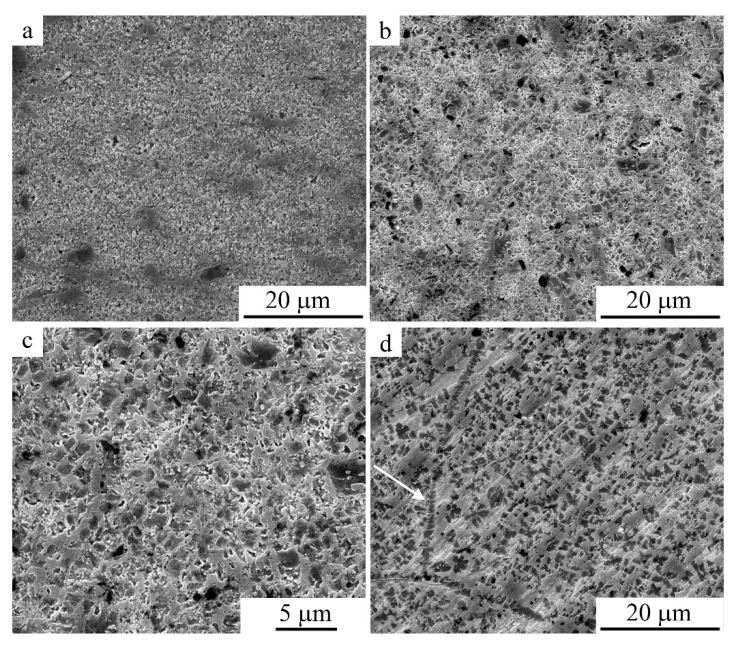
Secondary electron SEM images of the intragrain microstructure in the Cu-14Al-4Ni alloy after the compression tests at 673 K ((**a**)—*v* = 5, (**b**,**c**)—*v* = 1, (**d**)—*v* = 0.5 mm/min).

**Figure 8:**
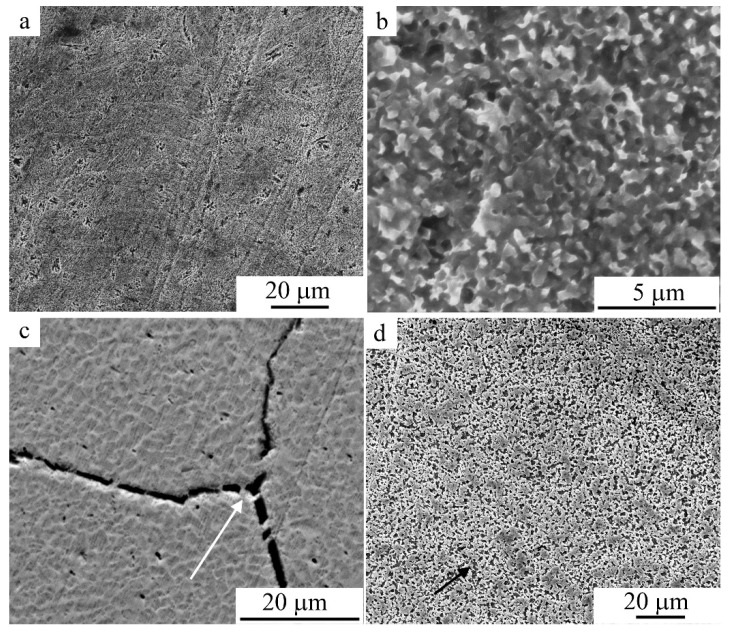
Secondary electron SEM images of the intragrain microstructure in the Cu-14Al-4Ni alloy after the compression tests at (**a**,**b**) 773 K (*v* = 1 mm/min) and (**c**,**d**) 873 K ((**c**)—*v* = 5, (**d**)—*v* = 1 mm/min).

The most typical images of the grain microstructure revealed during chemically selective electrolytic etching of an alloy that experienced dynamic recrystallization during MPC are also shown in Figure 6, Figure 7 and Figure 8 (the arrows in the figures shows grain boundaries decorated with γ_2_-phase particles). This is evidenced by the above images of the FG structure of the alloy with grains of 100–200 μm in size—an order of magnitude smaller than the grain sizes in the original CG alloy (Figure 6). At the same time, after MPC at 673, 773, and 873 K, the grains contained much smaller crystallites (1–2 μm in size), identified as α and γ_2_ phases (Figure 7 and Figure 8). Attention should be drawn to the fact that under MPC, the predominant pro-eutectoid heterogeneous decomposition of the γ_2_ phase along the boundaries of the austenitic grains preceded the homogeneous intragrain precipitation of the γ_2_ and α phases. At the same time, γ_2_ secretions, as is known, have a characteristic cuboid-pointed micromorphology, and α particles have an equiaxial shape on the structural images (Figure 7 and Figure 8).

**Figure 9:**
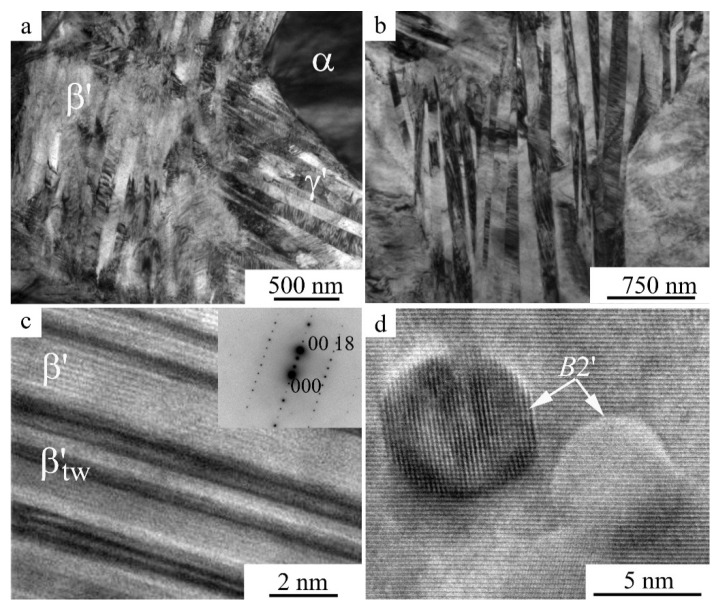
(**a**,**b**) Bright-field TEM images (amplitude contrast) and (**c**,**d**) high-resolution TEM images (phase contrast) of (**a**–**c**) the martensite microstructure and (**d**) *B*2′ phase particles after compression tests at (**a**,**c**) 873 K, (**b**) 973 K, and (**d**) 673 K.

Figure 9 shows TEM patterns of the martensite structure after MPC at (a) 873 and (b) 973 K, and (c,d) direct resolution of β′ martensite ((c), after MPC at 873 K) and two *B*2′ particles ((d), after MPC at 673 K).

**Figure 10:**
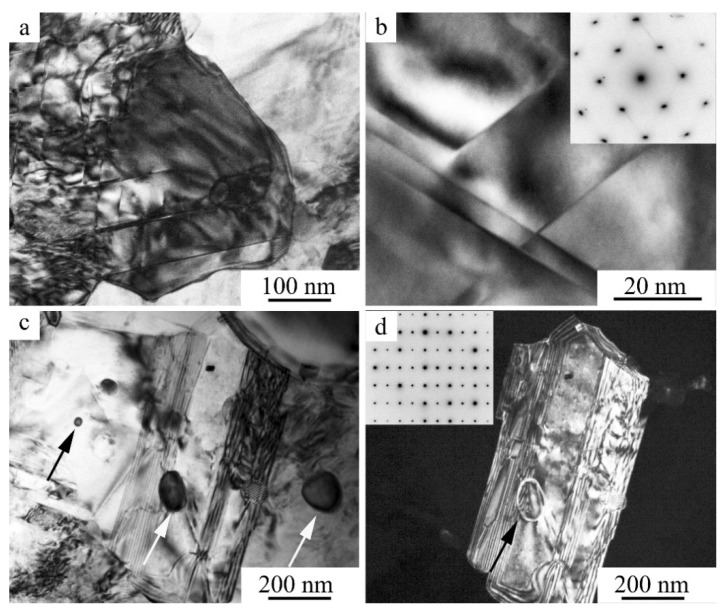
(**a**,**c**) Bright- and (**b**,**d**) dark-field TEM images (amplitude contrast) of the microstructure of the (**a**,**b**) α, (**c**,**d**) γ_2_, and *B*2′ phases after compression tests at 773 K (*v* = 1 mm/min).

Figure 10 shows TEM images of the fine structure of α, γ_2_, and *B*2′ particles after MPC at 773 K. As follows from their analysis, the grains and crystallites of the α phase formed inside the β_1_ matrix in the course of the compression tests experienced noticeable plastic deformation, with the formation of mesh–cellular dislocation and twin substructures (see Figure 10a,b), in contrast to the more brittle, solid, and often twinned γ_2_ phase (see Figure 10c,d). In addition, *B*2′ phase rounded precipitates based on the Ni-Al system were observed (shown by arrows in Figure 10c,d), which had much smaller dimensions (not exceeding 100 nm), and formed in the initial austenite, at the boundaries, and inside the α and γ_2_ inclusions. According to X-ray energy-dispersive elemental microanalysis, in agreement with the data [4], α crystals were somewhat depleted of aluminum (after the alloy’s offsetting at 673 and 773 K to 10 wt.%), and γ_2_ precipitations—along with copper—contained up to 5 at.% Ni, whereas *B*2′ precipitates—along with nickel and aluminum—contained up to 5 at.% Cu.

**Figure 11:**
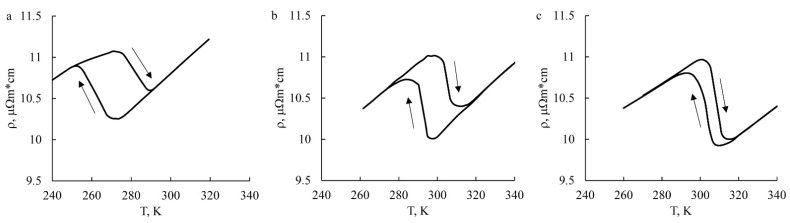
Temperature dependences ρ(*T*) of the Cu-14Al-4Ni alloy after (**a**) quenching, (**b**) MPC at 973 K, and (**c**) MPC at 773 K. The arrows show the heating and cooling during the experiment.

According to temperature measurements of electrical resistance ρ(*T*), it was found that the critical TMT temperatures of MPC alloys increased compared to the temperatures of the quenched alloy (Figure 11, Table 2). These temperatures became noticeably higher than room temperature due to some depletion of the alloy matrix by aluminum caused by the partial decomposition that occurred.

## 4. Discussion

Thus, firstly, we found that during isothermal compression, a dynamic recrystallization process took place in the alloy, as a result of which an FG austenite structure with a homogeneous grain size appeared, which was significantly more disperse in size than in the initial alloy. Secondly, during compression at 873 K and—to a lesser extent—at 973 and 1073 K, in the *D*0_3_ austenite, in accordance with the phase equilibrium diagram [4], partial pro-eutectoid decomposition was induced by the heterogeneous and homogeneous precipitation of the γ_2_ phase, the size of the precipitates of which increased with increasing compression duration. Meanwhile, at 673 and 773 K (that is, below *T*_ED_), along with the γ_2_ phase, the α- and *B*2′-phase particles were precipitated. The presence of precipitates of these phases after compression at 673 and 773 K was recorded in the alloy after all of the treatments used.

The size refinement of *D*0_3_ austenitic grains occurred as a result of dynamic recrystallization, which initially preceded the decomposition. Therefore, their boundaries were the predominant sites of heterogeneous nucleation, and led to subsequent decoration by precipitating phases. Their barrier effect, as is known, can restrain the subsequent growth of grains with continued compression. Upon cooling to RT after MPC, the austenite alloy experienced a TMT with the formation of the β′ and γ′ martensitic phases of packet morphology.

When testing the mechanical properties during hot MPC of the austenite at 973 and 1073 K, even higher strength values of the alloy were found (Figure 2d, Table 1). The alloy experienced fracture at high values of the ultimate strength σ_u_, having previously demonstrated a stage of significant steady uniform deformation ε_st_ (up to 50%) at a low value of the yield stress σ_y_ (less than 100 MPa), and then a strong deformation hardening at the next stage of plastic deformation up to failure ε_f_ (up to 70%). Apparently, during hot MPC, specific processes of continuous dynamic recrystallization took place under the influence of an external load, the value of which was initially small at the steady stage of plastic yielding, and then sharply increased (up to 2 GPa). This ensured the formation of an equiaxial FG structure in the initial CG alloy.

The results of subsequent tensile tests of mechanical properties of the alloy at RT are shown in Figure 2f. The properties of the FG alloy samples themselves were measured after MPC at 973 and 1073 K, as well as compared with the CG alloy in its initial state after forging at 1223 K and quenching (insert in Figure 2a). A unique feature of its mechanical behavior during tensile tests after MPC was the presence of a stage of martensitic inelastic pseudo-fluidity (ε_m_ ≤ 4%) at a low value of stress (σ_m_) at the start of the reorientation of the twin-martensitic structure in the direction of the acting tension force (σ_m_ < 100 MPa). Then, a strong deformation hardening occurred, culminating in the failure of the samples at high values of σ_u_ after significant total plastic deformation for these alloys (δ = 14–16%) (see Figure 2f). The curve in the insert in Figure 2a, on the other hand, illustrates the low strength and plastic properties of a conventional quenched CG austenitic alloy.

The results of the fractographic study of the fractures after tensile tests of FG alloys at RT are shown in Figure 4c,d for MPC at (c) 973 K and (d) 1073 K. Despite the sufficiently high plastic deformation of martensite, it can be concluded from the appearance of the fracture surfaces that the failure occurred simultaneously by tough–ductile and tough–ductile–brittle mechanisms. On the surface of the fractures of the tensile test samples after compression, there were cup-shaped fine-dimpled zones of tough–ductile fracture, flatter chips with a river-like internal pattern, and individual cracks along the boundaries of both grains and disperse martensite crystals (Figure 4e,f). However, in this case, the size of the flat elements on the rupture images was 5–10 μm—more than an order of magnitude smaller than the grain sizes (100–200 μm). This convincingly characterizes the origin of the formed subgrain structural elements responsible for the development of the mechanism of tough–ductile–brittle intragrain rupture. It is clear that the rupture could occur both via quasi-cleavage along the boundaries of the interface of disperse martensite packets, and by tough–ductile mechanisms across the crystals mainly oriented along the axis of tension.

Thus, the formation of a fine-grained structure caused an unusual combination of strength and plasticity of the initially brittle alloy, both under controlled uniaxial compression, and during subsequent tensile tests at room temperature. The discovered data evidently demonstrate the attractive industrial potential of eutectoid Cu-based alloys, due to the significant improvement in their strength and ductile characteristics.

## 5. Conclusions

Thus, in this work, the main regularities of the formation of mechanical properties and the regularities of structural-phase transformations of the Cu-14 wt.%Al-4 wt.%Ni alloy after controlled isothermal compression testing using a machine with different test rates (0.5; 1; 5 mm/min), in the temperature range from RT to 1073 K, as well as during tensile tests, were as follows:

1. Cold isothermal compression of the quenched alloy, without changing the grain sizes, ensured the formation of a fine crystalline structure of the packet martensite, and enhanced values of strength and plastic properties under compression conditions (σ_u_ = 1150 MPa, σ_y_ = 400 MPa, ε = 22%), due to the effective redistribution and adaptation of elastic volume and shear stresses over the volume of the alloy, caused by a deformation-induced oriented TMT.
2. It was found that when compressed at high temperatures (873–1073 K), the alloy in the austenitic state had a high ability to harden and, at the same time, to undergo plastic deformation (σ_u_ = 1550 ÷ 2000 MPa, σ_y_ = 380 ÷ 50 MPa, ε = 95 ÷ 58%). The discovered effect of plasticization under controlled high-temperature isothermal compression was due to the dynamic recrystallization of the alloy, with the formation of an FG austenite structure (with grain size up to 100–200 μm) capable of developed plastic deformation.
3. Isothermal compression at 673–873 K, along with the formation of a fine-grained (up to 100–200 μm) structure, leads to radical intragrain size refinement (UFG up to 1–5 μm) due to the mechanism of complex dynamic recrystallization at temperatures below *T*_ED_, together with the precipitation of highly disperse particles of the γ_2_, α, and *B*2′ Ni-Al-Cu phases.
4. The effective formation of a mixture of the FG and UFG structures in partially aged austenite during a complex reaction of dynamic recrystallization and pro-eutectoid (above 840 K) or eutectoid (below 840 K) decomposition caused an unusual combination of high strength and ductility of the deformable alloy, with a megaplastic deformation (ε in the range of 60–95%) and stresses at alloy failure (strength limit σ_u_), reaching 1600–2000 MPa. At the same time, after compression, a predominantly fine-dimpled tough–ductile intragrain rupture mechanism was obtained.
5. It was shown that the process of megaplastic compression was accompanied by the development of axial-deformation-induced recrystallization texture of the type <110>*_D_*_03_.
6. Subsequent cooling of the alloy after compression at elevated temperatures, without changing the UFG or FG structures, induced single-packet TMT. It can be assumed that this combined result provided a homogeneous distribution of disperse pairwise-twinned martensitic crystals over the volume of the alloy, along with a favorable mutual accommodation of elastic volume and shear stresses, due to the TMT.
7. The rupture (failure) of the FG alloy in the martensitic state under uniaxial tension occurred after the stages of phase yielding and, thereafter, significant hardening due to the developed plastic deformation, which was completed by the action of the fine-dimpled tough–ductile and tough–ductile–brittle intragrain fracture mechanisms (operating along the boundaries of disperse martensite crystals), thereby causing the increased strength and plasticity of the alloy in the martensitic state (with σ_u_ = 1100–1600 MPa, σ_m_ = 80 MPa, ε_m_ = 2÷3%, and δ = 14–16%).

## Figures and Tables

**Table 1:** Mechanical characteristics of the Cu-14Al-4Ni alloy after MPC at various temperatures and rates (*v*).

Treatment	σ_y_, MPa	σ_u_, MPa	ε, %	θ_1_, GPa	θ_2_, GPa
RT, *v* = 1 mm/min	400	1150	22	3.5	-
673 K, *v* = 0.5 mm/min	360	1550	82	0.7	5.3
673 K, *v* = 1 mm/min	380	1550	84	0.7	5.7
673 K, *v* = 5 mm/min	530	1580	76	0.2	4.6
773 K, *v* = 1 mm/min	250	1550	84	0.3	6.3
773 K, *v* = 5 mm/min	310	1620	83	0.3	5.9
873 K, *v* = 1 mm/min	70	1550	95	0.2	8.0
873 K, *v* = 5 mm/min	120	1550	92	0.1	8.1
973 K, *v* = 1 mm/min	50	2000	70	0.1	10.5
1073 K, *v* = 1 mm/min	50	2000	55	0.1	11.0

**Table 2:** Critical temperatures of the start (*M_s_*, *A_s_*) and finish (*M_f_*, *A_f_*) of the forward and reverse TMTs in the Cu-14Al-4Ni alloy after various treatments.

Treatment	*M_s_*, K	*M_f_*, K	*A_s_*, K	*A_f_*, K	Δ*T* *, K
Quenching	270	250	275	290	22
MPC, 973 K	295	285	300	310	15
MPC, 773 K	305	290	300	315	10

* Δ*T* = 1/2[(*A_s_* + *A_f_*) − (*M_s_* + *M_f_*)].

## Data Availability

All data included in this study are available upon request by contact with the corresponding author.
